# A Comparative Evaluation of Flexural and Impact Strength of Polymethyl Methacrylate (PMMA) Reinforced With Graphene and Multi-Walled Carbon Nanotubes: An In Vitro Study

**DOI:** 10.7759/cureus.40024

**Published:** 2023-06-06

**Authors:** Neeranshi Swaroop, Pratibha Katiyar, Kaushik Kumar Pandey, Fauzia Tarannum, Mohd Umar, Swarupa Paul, Nikhil V. Dayama, Asra Ahmed, Ragini Singh

**Affiliations:** 1 Prosthodontics, Career Post Graduate Institute of Dental Sciences & Hospital, Lucknow, IND

**Keywords:** impact strength, flexural strength, multiwalled carbon nanotubes, graphene, heat cure polymethyl methacrylate

## Abstract

Background and objective

Low flexural strength (FS) and impact strength (IS) are major drawbacks in removable prostheses made from polymethyl methacrylate (PMMA). Attempts to enhance the strength and longevity of these prostheses have been of keen interest among researchers. Nanofillers are new and advanced reinforcements that can chemically modify PMMA. Graphene and multi-walled carbon nanotubes (MWCNTs) were used in this study to evaluate FS and IS when added to polymer and monomer individually.

Method

Four groups were created based on the addition of nanofillers: no nanofillers - control; 0.5% by weight of graphene; 0.5% by weight of MWCNT; and 0.25% by weight of both. These groups were further subdivided into two according to the nanofiller being added to polymer and monomer each. The samples were then subjected to a 3-point bending test to assess FS, and an Izod impact tester was used to test IS.

Results

Decreased FS and FS were seen in all groups with the addition of nanofillers in the polymer (p<0.001). With the addition of nanofillers in monomer, increased FS and IS were seen in groups with MWCNTs whereas a decrease was seen with the addition of graphene (p<0.001).

Conclusion

Nanofillers should be added to the monomer of heat-cure PMMA instead of polymer; 0.5% by weight of MWCNT has shown the highest FS and IS when added to the monomer.

## Introduction

Removable prostheses are created for patients who are fully or partially edentulous, by using various materials such as wood, ivory, vulcanite, stainless steel, etc. Polymethyl methacrylate (PMMA), invented by Dr. Walter Wright in 1937 [1], is one of the most widely used denture-base materials in the manufacturing of complete dentures, with decades of research supporting its use. PMMA has been successfully utilized as a denture base material due to its low cost, ease of production, acceptable aesthetics, biocompatibility, low water sorption, and adequate strength [2].

However, polymeric products do not show optimum mechanical strength in various situations. Early cracks and fractures have been frequently observed in dentures due to their mechanical properties [3]. Two crucial characteristics that give strength and longevity to prostheses are flexural strength (FS) and impact strength (IS) [1]. A prosthesis may break inside or outside the mouth, due to continuous wear or from extra-oral high-impact force brought on by accidentally dropping the prosthesis. High IS and FS are desirable attributes for PMMA to reduce the risk of fracture [2].

The field of acrylics has undergone significant new developments to address these issues and improve the longevity and durability of prostheses. There has been a great interest in strengthening denture base materials with metal mesh, glass fibers, etc [2]. Recently, the process of incorporating filler particles into resin to enhance its qualities by chemical modification of PMMA has received immense interest. Two nanofillers employed as reinforcement materials are graphene and multi-walled carbon nanotubes (MWCNTs). Graphene has the basic construction of graphite, which consists of carbon atoms arranged in a honeycomb structure in the form of flat thick sheets. Its derivatives can achieve good dispersion within different polymers and can be easily processed [2]. Carbon nanotubes (CNTs) are strong, resilient, and lightweight, and usually form stable cylindrical structures. The addition of carbon fibers to a matrix gives strength and elasticity to the material and also improves its toughness. It can successfully reinforce the fracture lines by strengthening the fibrils and bridging voids [4].

In light of this, the present in vitro study was designed to evaluate and compare the mechanical properties such as FS and IS of heat-polymerized PMMA reinforced with graphene and MWCNTs.

## Materials and methods

**Figure 1:**
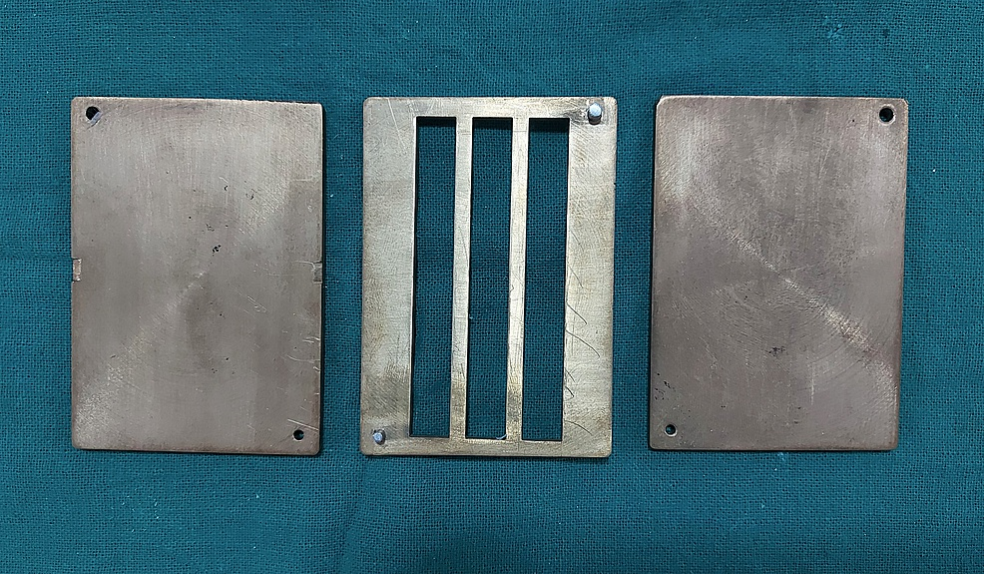
Custom fabricated brass die of 12 x 6 cm with three slots of 80 x 10 x 4 mm

The present in vitro study was conducted in the Department of Prosthodontics and Crown & Bridge, Career Post Graduate Institute of Dental Sciences & Hospital, Lucknow, and the testing of samples was done at the Central Institute of Plastic Engineering and Technology, Amausi, Lucknow. A total of 80 specimens with a dimension of 80 x 10 x 4 mm (ISO 20795) [2] were fabricated for this study by using a custom-made brass die (Figure 1).

A total of 80 specimens were divided into two groups comprising 40 samples each: Group 1, in which nanofillers were added to the polymer; and Group 2, in which nanofillers were added to the monomer. Both groups were further subdivided into four groups with 10 samples each, based on the type and amount of nanofiller added.

Group 1 was subdivided into (i) Group Ap (control) - heat-cure PMMA polymer without any nanofiller; (ii) Group Bp - heat-cure PMMA polymer with 0.5% by weight of graphene; (iii) Group Cp - heat-cure PMMA polymer with 0.5% by weight of MWCNT and (iv) Group Dp - heat-cure PMMA polymer with 0.25% by weight of graphene and 0.25% by weight of MWCNT (Table 1). Group 2 was subdivided into (i) Group Am (control) - heat-cure PMMA monomer without any nanofiller; (ii) Group Bm - heat-cure PMMA monomer with 0.5% by weight of graphene; (iii) Group Cm - heat-cure PMMA monomer with 0.5% by weight of MWCNT; and (iv) Group Dm - heat-cure PMMA monomer with 0.25% by weight of graphene and 0.25% by weight of MWCNT (Table 1).

**Table 1:** Division of two groups into four subgroups based on the addition of nanofillers in either polymer or monomer

Group 1 - nanofillers added in the polymer					
	Group Ap	Group Bp	Group Cp	Group Dp	Total
No. of samples	10	10	10	10	40
Group 2 - nanofillers added in the monomer					
	Group Am	Group Bm	Group Cm	Group Dm	Total
No. of samples	10	10	10	10	40

**Figure 2:**
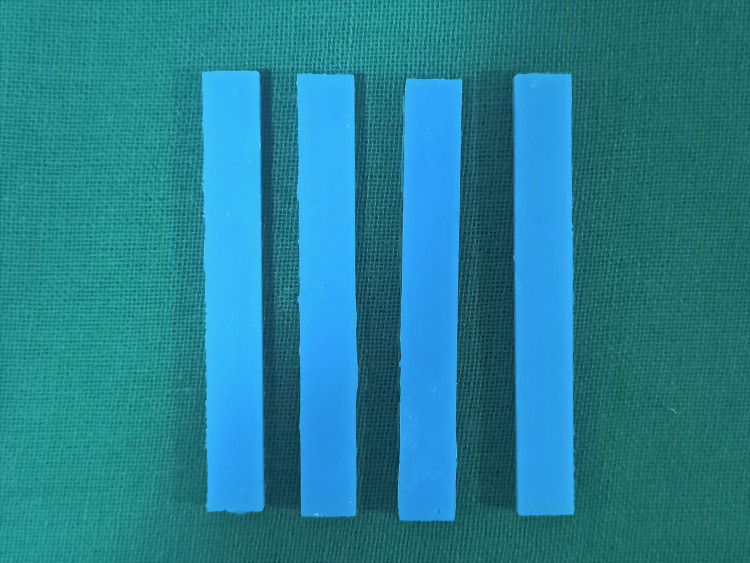
Polyvinyl siloxane die retrieved from the custom-made brass metal die with a dimension of 80 x 10 x 4 mm

Duplication of the metal die dimensions was done by using polyvinyl siloxane [Elite Double 32 fast duplicating material (Zhermack Technical, Rovigo, Italy)], and the mold for specimens was prepared. The duplicating material was mixed in equal proportions and loaded into the custom brass die (Figure 2).

The polyvinyl siloxane die was then invested in gypsum product type 2 (dental plaster) and type 4 (die stone) with a two-pour technique. After the second pour was set, the two halves of the flask were opened, and the polyvinyl siloxane dies were gently removed, thus making the molds for the fabrication of specimens. Nanofillers were measured for addition to heat-cure acrylic resin polymer and monomer by using an electronic weighing scale (MH-Series). For Group Bp and Bm specimens, 0.5 gm graphene was measured (Ad-Nano Technologies, Shimoga, India). For Group Cp and Cm specimens, 0.5 gm MWCNTs were measured (Ad-Nano Technologies). For Group Dp and Dm specimens, 0.25 gm graphene and 0.25 gm MWCNT were measured.

**Figure 3:**
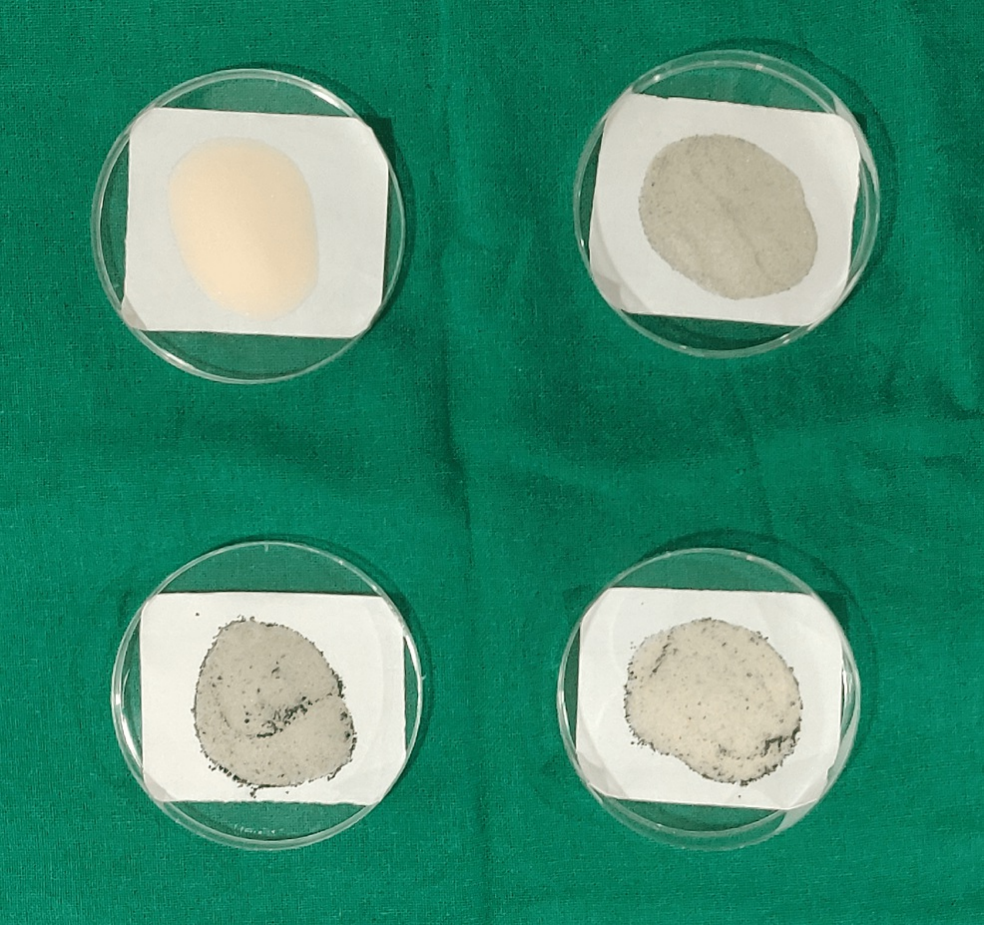
Measuring the weight of nanofillers mixed in the polymer of heat-cure PMMA with the help of a vibrating device PMMA: polymethyl methacrylate

Then, 100 gm of the polymer was mixed with each type of nanofiller in a vibrating device [5,6] to make 10 samples each of Group Ap, Bp, Cp, and Dp for measuring FS and IS (Figure 3).

**Figure 4:**
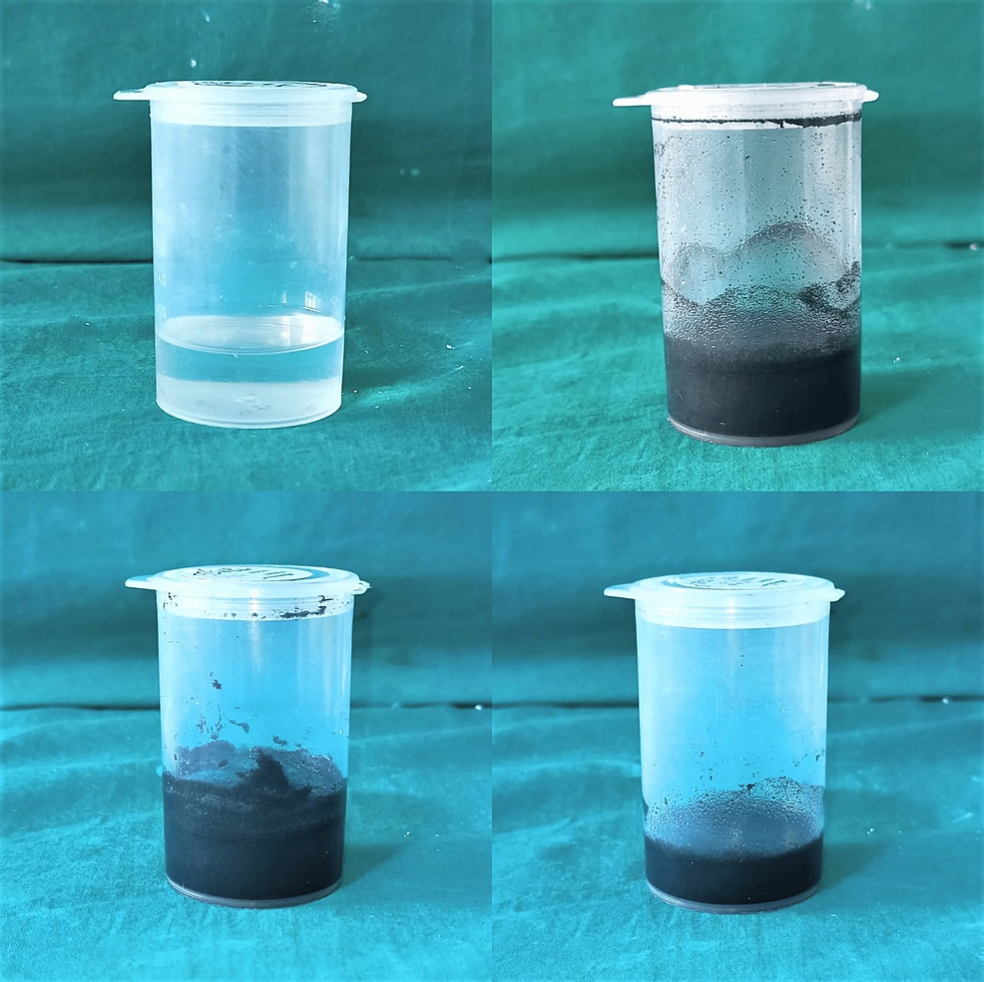
Measuring the weight of nanofillers mixed in the monomer of heat-cure PMMA in an ultrasonic mixing unit PMMA: polymethyl methacrylate

Similarly, the measured nanofillers were added to 106 ml (99.5% by weight) of methyl methacrylate monomer measured in a beaker to make 10 samples each of Group Am, Bm, Cm, and Dm for measuring FS and IS. The settling tendency of the nanofillers was overcome by agitating the solution in an ultrasonic unit for 15 minutes to ensure homogenous dispersion (Figure 4) [7].

**Figure 5:**
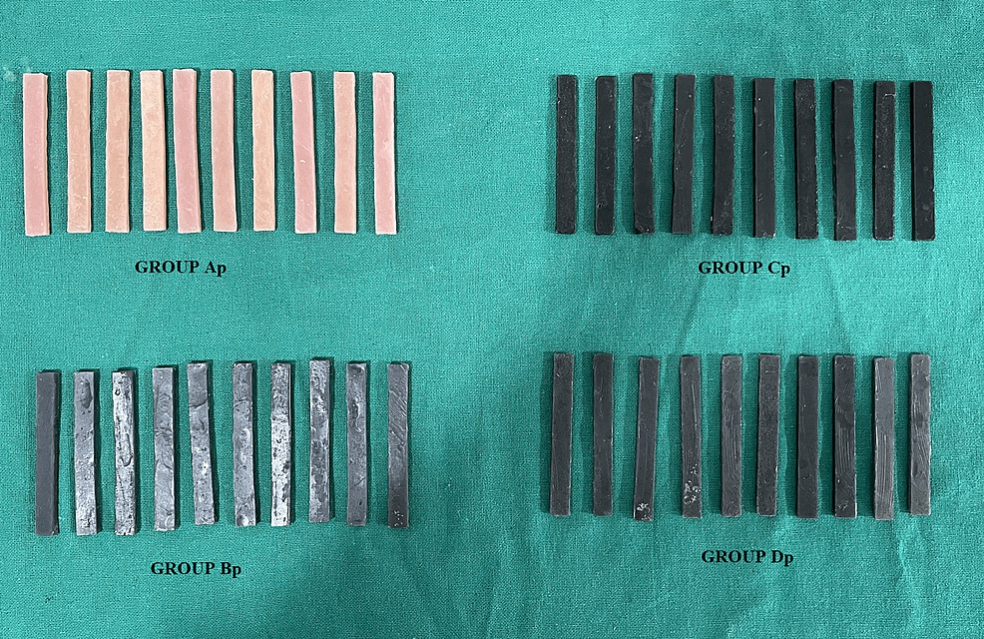
Specimens fabricated after the addition of nanofillers to the polymer of heat-polymerized PMMA PMMA: polymethyl methacrylate

To fabricate all the specimens, measured heat-cure polymer was mixed with monomer in a standard ratio of 1:2 by weight. The resin dough was packed in the stone mold. The specimens were left for bench-curing for 30 minutes, after which a short curing cycle was performed for polymerization. The specimens were allowed to bench-cool before deflasking. The specimens formed after the addition of nanofiller in the polymer were retrieved, trimmed, and finished (Figure 5).

**Figure 6:**
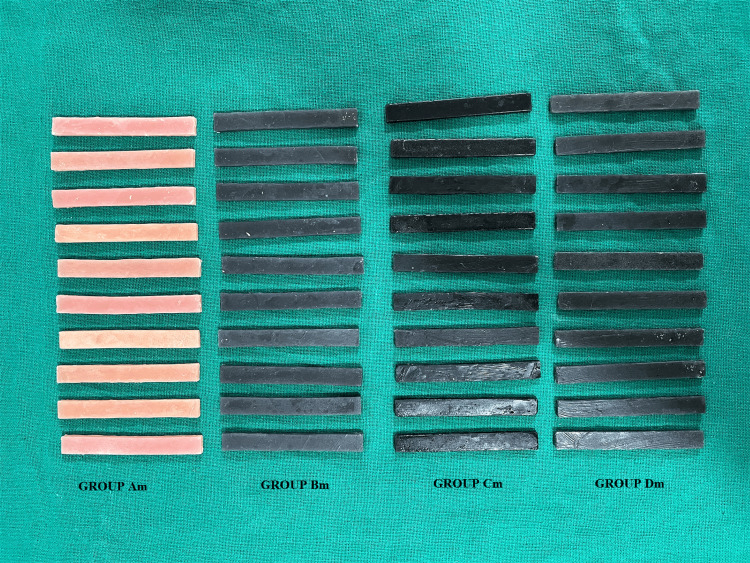
Specimens fabricated after the addition of nanofillers to the monomer of heat-polymerized PMMA PMMA: polymethyl methacrylate

Specimens formed after the addition of nanofillers to monomer were also retrieved, trimmed, and finished in a similar fashion (Figure 6).

**Figure 7:**
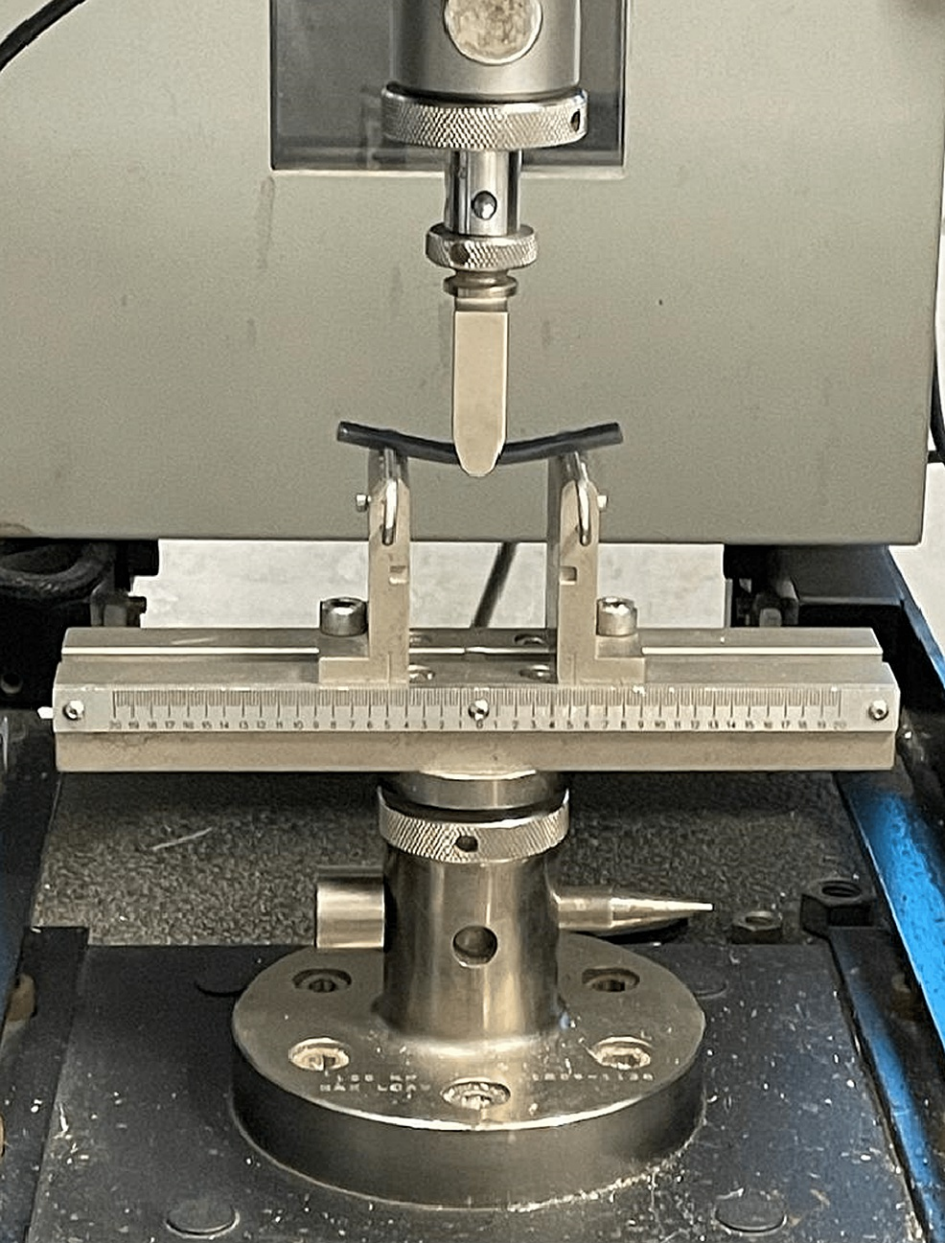
Specimens tested for flexural strength in the digital universal testing machine

To measure FS, a digital universal testing machine (Instron 3382, Central Institute of Plastic Engineering and Technology, Amausi, Lucknow, India) was used. The distance between the specimen supports was 40 mm, and the loading force applied to the specimens was at a crosshead speed of 5 mm/minute until the specimens fractured. Readings were recorded in megapascals (MPa) (Figure 7).

**Figure 8:**
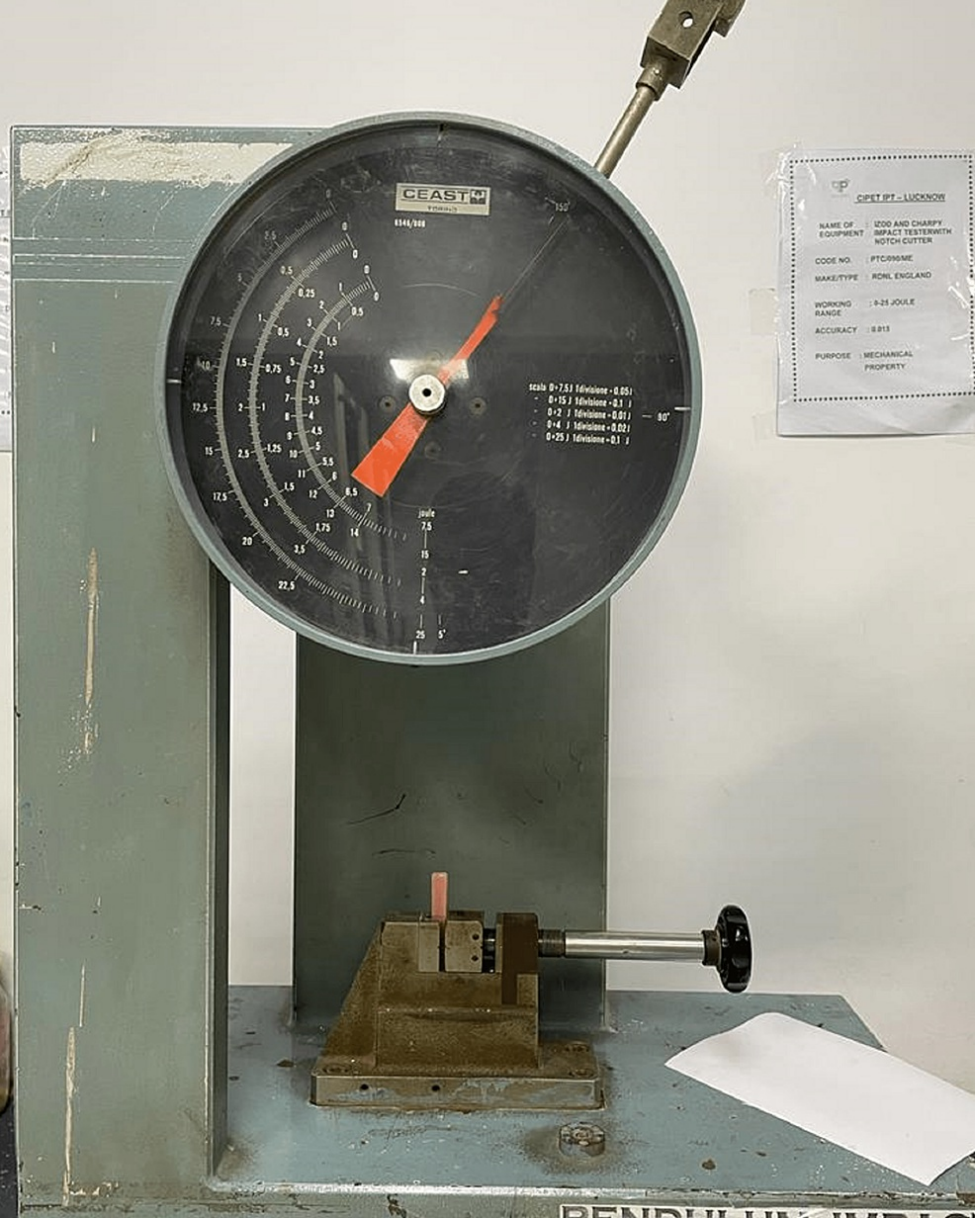
Specimens placed in the slot for measuring impact strength in the Izod impact tester

The IS test was conducted using the Izod impact testing machine (Central Institute of Plastic Engineering and Technology) and readings were recorded in kilojoules per square meter (kJ/m^2^) (Figure 8).

## Results

The mean FS was the highest for Group Ap, in which no nanofiller was added to the polymer, i.e., 76.23 MPa, followed by Group Cp, Group Dp, and Group Bp. The mean IS, when nanofillers were added to the polymer was the highest for Group Ap, i.e., 12.1918 kJ/m^2^, followed by Group Cp, Group Dp, and Group Bp. The difference in these values, when nanofillers were added to the polymer of heat-cure PMMA, was statistically significant (p<0.001) (Table 2).

**Table 2:** Comparison of mean flexural strength and impact strength of four groups after the addition of nanofillers in the polymer as evaluated by ANOVA *Statistically significant ANOVA: analysis of variance

Strength	Group	Mean	Standard deviation	F	P-value
Flexural strength, MPa	Group Ap	76.2300	0.18708	2682.950	<0.001*
	Group Bp	56.2540	0.38734		
	Group Cp	65.4340	0.44948		
	Group Dp	59.5300	0.43261		
Impact strength, kJ/m^2^	Group Ap	12.1980	0.18939	115.036	<0.001*
	Group Bp	8.0940	0.22367		
	Group Cp	9.8260	0.62532		
	Group Dp	8.5180	0.34010		

When nanofillers were added to the monomer, the mean FS of Group Cm, in which 0.5% by weight of MWCNT was added to the monomer, was the highest i.e., 80.680 MPa, followed by Group Dm, Group Am, and Group Bm. The mean IS for the same samples was the highest for Group Cm, i.e., 14.3060 kJ/m^2^, followed by Group Dm and Group Am, with the lowest readings observed in Group Bm, i.e., 9.7100 kJ/m^2^. The difference in the values of FS and IS when nanofillers were added to the monomer of heat-cure PMMA was statistically significant (p<0.001) (Table 3).

**Table 3:** Comparison of mean flexural strength and impact strength of four groups after the addition of nanofillers in the monomer as evaluated by ANOVA *Statistically significant ANOVA: analysis of variance

Strength	Group	Mean	Standard deviation	F	P-value
Flexural strength, MPa	Group Am	76.1460	0.24906	120.840	<0.001*
	Group Bm	73.5360	0.49581		
	Group Cm	80.6800	0.97691		
	Group Dm	78.4360	0.54049		
Impact strength, kJ/m^2^	Group Am	12.2080	0.29287	49.026	<0.001*
	Group Bm	9.7100	0.70310		
	Group Cm	14.3060	0.61954		
	Group Dm	13.1180	0.76607		

## Discussion

PMMA is the chemical ester of either acrylic acid or methacrylic acid (MMA). MMA/PMMA is usually supplied as powder and liquid components, the mixing of which is done by the dissolution of polymer powder in the monomer form, a kneadable dough-like mass. The polymerization process is either heat-activated, chemically activated, or light-activated [8]. Heat-activated PMMA is widely used in prosthodontics for the fabrication of various types of removable prostheses due to its salient characteristics: it is plastic and hence can be molded to the required shape and form; easy to manipulate and process; biocompatible and hence has dimensional stability in the oral environment; lightweight; and economical [9]. Despite all these advantages, the fracture of the prosthesis is very common. This is attributed to its mechanical properties. Due to low FS, repeated flexing of material occurs when subjected to loads. This leads to stress concentration developing microcracks, which further intensifies and leads to midline fracture of the denture [10]. Repair of fracture or reinforcing the heat-cure PMMA with different materials has been suggested to increase the FS and IS. Chemical and mechanical reinforcements in the form of metal mesh, as well as the addition of other fibers like glass, carbon, Kevlar, sapphire, polyester, nylon, etc., have all been tried in order to enhance FS [11,12].

Nanoparticles have also been increasingly used in material science due to their enhanced properties. This has led to the chemical modification of PMMA with nanofillers to improve its mechanical properties. The nanofillers used in different studies and experiments to reinforce PMMA include nanoparticles of metal oxides (ZrO_2_, TiO_2_, Al_2_O_3_) [13,14], as well as other metals and non-metals such as silver, silica, and carbon. This modification is based on the high interfacial shear strength between the nanofiller and resin matrix, resulting from cross-links formation or supra-molecular bonding that covers the nanofiller, which in turn prevents the propagation of cracks [15]. Graphene and CNTs have superior physical, chemical, and thermal properties and have been used as reinforcement fillers in PMMA at different concentrations. Multiple studies have evaluated the effect of adding graphenes and CNTs on thermal conductivity, strength, antibacterial activity, and other physical and chemical properties of PMMA. Nanofillers have been added in different ratios by various methods, generally in auto-polymerized PMMA, in major studies [16,17].

Graphene, a fundamental form of graphite composed of carbon atoms, is organized in a honeycomb structure in the form of thick, flat sheets [1]. It performs better than other traditional nanofillers due to its high surface area, tensile strength, flexibility, and low coefficient of thermal expansion. Graphene oxide (GO) is chemically favored to enhance its matrix compatibility [18]. Due to improved mechanical, physical, and chemical properties, CNTs are resilient, lightweight materials with a significant potential for biological applications. They are hollow and cylindrical in shape, with walls made of hexagonal carbon rings. They exist in two forms: single-walled carbon nanotubes (SWCNTs) and MWCNTs [19]. The addition of these nanotubes gives strength and improves toughness because of their higher intrinsic strengths and moduli, showing good dispersion and good adhesion to polymers, indicating chemical compatibility and the creation of chemical bonds between the surface and polymer [4].

In the present study, 0.5% by weight of the powder form of graphene nanofiller was mixed in heat-cure PMMA polymer (Group Bp) by using a vibrating lab device [16]. A highly significant (p<0.001) decrease in FS and IS was observed in this group of specimens (Table 2). Bacali et al. [5] have observed that FS increased three to four-fold in their study. The reason for the difference found could be due to decreased concentration of nanofiller added into heat-cure PMMA in the present study instead of auto-polymerized PMMA. Furthermore, graphene was used in oxide form instead of in combination with Ag nanoparticles, leading to a non-homogenous mix of powder [20]. The same concentration of 0.5% by weight of graphene was also added to the heat-cure PMMA monomer (Group Bm), which underwent agitation in an ultrasonic mixing unit [7]. Comparison of these samples with the control group (Group Am) showed highly significant (p<0.001) decreased values of FS and IS (Table 3). Similar results were found by Ghosh and Shetty [7] in their study. This may be because graphene sheets tend to aggregate and become large enough to provide steric obstacles that stop further chemical reactions. This limits the flow of the polymer into the solution, causing voids to form, increasing stress concentrations, and making it prone to fracture [7]. Swami et al. [2] and Khan et al. [21] came to the conclusion that the same concentration of graphene increased FS and IS. The difference may have been caused by the addition of graphene oxide sheets and solution-form nanofillers to the monomer of cold-cure acrylic resin, as opposed to heat-cure PMMA as in our study.

In this study, 0.5% by weight of MWCNT was added to the polymer of heat-cure PMMA (Group Cp) in a similar manner [6]. Comparing the specimens of Group Cp with the control (Group Ap), a highly significant (p<0.001) decrease in FS and IS values was observed (Table 2). The possible reason for such findings could be the difference in particle size of polymer and nanotubes and hence the improper mixing of both powders. Consequently, the polymerization reaction between modified polymer particles and monomer could not complete, and led to void formation, making it prone to fracture. The same concentration of 0.5% MWCNT was added to the heat-cure PMMA monomer (Group Cm) and a solution was formed after ultrasonic mixing [7]. When this monomer was used to make specimens and compared with the control group (Group Am), FS and IS values were highly significantly increased (p<0.001) (Table 3). The findings were in line with those of research by Ghosh and Shetty [7], Swami et al. [2], and Wang et al. [4]. The main variation between Ghosh and Shetty's and Swami et al.'s investigations was the addition of an auto-polymerized acrylic resin monomer. CNTs are effective in bridging the cracks. Their spatial hexagonal ring configuration reduces segmental motion, which gives the mixture strength and stability. Due to their strong dispersibility, these CNTs may fill the gaps between polymer chains, which boosts strength and rigidity while preventing chain movement. Wang et al. [4] introduced MWCNTs to the monomer of heat-cure PMMA by ultrasonic mixing in different concentrations. The highest rise in FS was observed at 0.5% concentration. For the 2% MWCNT-PMMA group, there was a minor drop in FS. This was attributed to non-homogenous dispersion when 2% CNTs were used, which led to more agglomerations and micro-voids. Moreover, any load applied to the polymer matrix should be passed to the nanotubes, preferably with strong interfacial bonding between PMMA and CNTs, as is the case with 0.5% and 1% concentrations.

In this study, a combination of both nanofillers (0.25% by weight of graphene and 0.25% by weight of MWCNT) was mixed with heat-cure PMMA polymer (Group Dp). The samples made were compared with the control group (Group Ap), which showed a statistically highly significant (p<0.001) decrease in FS and IS (Table 2). These findings could be due to differences in particle size, leading to a non-homogeneous mixture and improper polymerization [6]. The same combination of nanofillers (0.25% by weight of graphene and 0.25% by weight of MWCNT) was also added to the monomer (Group Dm) of heat-cure PMMA and compared to the control group (Group Am). The result showed a highly significant (p<0.001) increase in FS and a statistically significant (p=0.03) increase in IS (Table 3). The result is in line with a study conducted by Swami et al. [2] on cold-cure acrylic resin.

The decreased FS and IS in all groups may be due to improper mixing of these particles because of differences in particle size. The inherent ability of MWCNTs to increase strength and toughness may be responsible for the reduced mean difference when nanofillers were added to polymers. When comparing all groups in which nanofiller was added to the monomer of heat-cure PMMA, a significant increase was seen in FS and IS of two groups (Group Cm and Dm), while a decrease was seen in Group Bm. MWCNTs enhanced the physical properties when added to monomer due to their good dispersibility and better cross-linking, whereas graphene forms agglomerate, preventing further reaction and leading to void formation.

Limitations of the study

The unappealing color of both nanofillers is a major limitation. The dark grayish color of the material restricts their use in the visible zone of dentures. Further research on appropriate color alteration by adding a functional group to the nanofillers can still be conducted. SWCNTs are transparent, and hence their usage can be further studied to incorporate as a reinforcing material. The reduction in the quantity of nanofillers can also be examined. Further studies and investigations are needed to improve the dispersion of these nanofillers and effectively allow them to be used in multiple situations. Nonetheless, PMMA reinforced with these nanofillers may be easily employed in less noticeable regions such as the maxillary palate, mandibular lingual flange in instances of single full dentures, acrylic backing in maxillofacial prosthesis, and cranioplasts until these problems are resolved [7].

## Conclusions

Within the limitations of the study, we have reached the following conclusions based on our findings: nanofillers should be added and mixed with the monomer of heat-cure PMMA instead of polymer for better dispersion and cross-link formation; 0.5% by weight of MWCNT was the most effective nanofiller to be used for reinforcing heat-cure PMMA, as it has shown the highest FS and IS when added in the monomer. The unappealing color of both nanofillers presents a challenge, which requires further explorations for potential modifications. However, in the meantime, they can be utilized in the non-visible zone of dentures and for characterization in patients with darker complexion. To validate these findings and to identify a material with the desired qualities for therapeutic success, further in vitro research using these materials is recommended.
